# A systematic literature review of the quality of evidence for injury and rehabilitation interventions in humanitarian crises

**DOI:** 10.1007/s00038-015-0723-6

**Published:** 2015-08-23

**Authors:** James Smith, Bayard Roberts, Abigail Knight, Richard Gosselin, Karl Blanchet

**Affiliations:** Public Health in Humanitarian Crises Group, London School of Hygiene and Tropical Medicine, London, UK; ECOHOST-The Centre for Health and Social Change, The London School of Hygiene and Tropical Medicine, London, UK; Department of Orthopaedic Surgery, University of California, San Francisco, USA

**Keywords:** Injury, Rehabilitation, Global surgery, Humanitarianism, Disasters, Conflict

## Abstract

**Introduction:**

Humanitarian crises continue to pose a significant threat to health; the United Nations estimates that 144 million people are directly affected by conflict or environmental disasters. During most humanitarian crises, surgical and rehabilitative interventions remain a priority.

**Objectives:**

This review assessed the quality of evidence that informs injury and physical rehabilitation interventions in humanitarian crises.

**Methods:**

Peer-reviewed and grey literature sources were assessed in a systematic manner. Selected papers were evaluated using quality criteria based on a modified version of the STROBE protocol.

**Results:**

46 papers met the inclusion criteria. 63 % of the papers referred to situations of armed conflict, of which the Yugoslav Wars were the most studied crisis context. 59 % of the studies were published since the year 2000. However, only two studies were considered of a high quality.

**Conclusions:**

While there is now a greater emphasis on research in this sector, the volume of evidence remains inadequate given the growing number of humanitarian programmes worldwide. Further research is needed to ensure a greater breadth and depth of understanding of the most appropriate interventions in different settings.

**Electronic supplementary material:**

The online version of this article (doi:10.1007/s00038-015-0723-6) contains supplementary material, which is available to authorized users.

## Introduction

Humanitarian crises continue to pose a significant threat to health. In 2012, the United Nations identified 144 million people directly affected by conflict or environmental disasters (OCHA 2013). During the acute phase of most humanitarian crises, the provision of surgical support remains a priority (Sphere Project 2011). A surge in the number of traumatic injuries in the acute phase can overwhelm pre-existing health services; for example, over a 10-week period following the 2010 Haiti earthquake, Médecins sans Frontières/Doctors without Borders (MSF) alone performed more than 4000 surgical procedures (Chu et al. 2011). At the same time, there is often a need to supplement routine surgical activities in the wake of widespread infrastructural damage and disruption to the local medical human resource pool. Rehabilitation interventions play an equally important role as efforts are made to support patients during their longer term recovery.

In recent years, increased scrutiny of the humanitarian sector has encouraged a drive towards professionalism and accountability, and has prompted humanitarian agencies to better demonstrate the impact and effectiveness of their programmes (Bradt 2009; Bantavala and Zwi 2000). Despite the fact that the treatment of injuries and the provision of rehabilitative programmes represent a key component of the health response during most humanitarian crises, the evidence base for these interventions is not well understood.

In an effort to better inform policy makers, donors, and other humanitarian stakeholders, the Wellcome Trust and the UK. Department for International Development (DfID) launched the Research for Health in Humanitarian Crises (R2HC) initiative in 2013. A systematic review was commissioned by R2HC to examine the quality and quantity of evidence for a range of contextual factors and the following health topics: communicable disease control; mental health and psychosocial support; sexual and reproductive health and gender-based violence; nutrition; water, sanitation and hygiene; non-communicable diseases; and injury and physical rehabilitation (Blanchet et al. 2013).

## Methods

Staff at the London School of Hygiene and Tropical Medicine (LSHTM) performed a systematic review of the available evidence for injury and physical rehabilitation interventions in crisis contexts. This review offers a thorough assessment of the quantity and quality of published evidence that informs humanitarian health programming in this field.

### Inclusion and exclusion criteria

Studies were selected or excluded based on the seven categories listed in Table 1. For the purpose of this review, we were concerned with health interventions in low- and middle-income countries only, as crises in these countries often present unique challenges that are not reproducible in high-income contexts. Similarly, interventions led by military contingents deployed from high-income countries for the treatment of injured combatants were not included in this review. While acknowledging that military medicine has advanced our understanding of the treatment of conflict-related injuries, the resources and facilities available to the military invariably create a unique environment that is unrepresentative of the broader crisis context.

**Table 1:** Inclusion and exclusion criteria

Category	Included	Excluded
Populations of interest	Populations affected by humanitarian crises and receiving humanitarian assistance (including refugees and internally displaced persons), in low- and middle-income countries (based upon World Bank country classification of 2012 (World Bank 2015)	Studies related to health interventions in high-income countries; studies pertaining to military operations involving combatants from high-income countries
Humanitarian crises	Studies that occurred during the acute, chronic, early recovery, or stabilisation phases of humanitarian crises including those that measured the impact of preparedness and resilience on public health outcomes during a humanitarian crisis	Studies that occurred before a humanitarian crisis (i.e. focused on preparedness or resilience measures), or that measured an outcome or intervention of interest in a post-crisis context
Intervention type	Public health interventions in which the outcome was measured before and after the intervention, or an intervention was studied against another intervention or control group	Studies with no specific health intervention (i.e. studies examining only health needs, prevalence, health risk factors, and coordination)
Health outcomes and outputs of interest	Primary outcomes (e.g. morbidity, mortality, vaccination status), secondary outcomes (e.g. attendance at health clinics, adherence to treatment)	Primary outputs (e.g. number of operations performed, number of surgical kits distributed, etc.)
Study design	Primary quantitative studies including: randomised and non-randomised controlled trials, longitudinal, cross-sectional, and economic studies	Qualitative studies (i.e. focused on processes and the perception of interventions); quantitative studies that did not measure a change in health outcomes; review papers
Intervention/publication date	January 1, 1980–April 30, 2013.	Studies published before 1980
Publication language	English, French	Any other language

This review sought to investigate outcomes attributed to interventions performed in acute and prolonged crises, the early recovery or stabilisation phases, or studies that examined a link between pre-emptive interventions and their effect on health outcomes following the onset of a crisis. For this reason, interventions in stable contexts were excluded. Studies were only considered eligible for inclusion if they documented that an intervention had taken place, and that subsequently either primary or secondary health outcomes had been measured. Any studies that documented outputs, but that did not draw an association between outputs and outcomes were excluded.

### Data sources

Both peer-reviewed and grey literature sources were evaluated. Peer-reviewed databases included: Embase, Medline, PsycInfo, International Bibliography of the Social Sciences (IBSS), and Global Health. The grey literature sources were chosen following consultation with specialists in the field of injury and rehabilitation, and included: SourceInfo, the International Disability and Development Consortium (IDDC), Leonard Cheshire Disability (LCD), ELDIS, European Disability Forum (EDF), Christoffel Blinded Mission (CBM), the Center for International Rehabilitation Research Information and Exchange (CIRRIE), Research for Development (R4D), MSF (Médecins Sans Frontières/Doctors Without Borders) France and Belgium, the Active Learning Network for Accountability and Performance (ALNAP), the World Health Organisation Library Database (WHOLIS), the Centre for Research on the Epidemiology of Disasters (CRED), and the International Society of Physical and Rehabilitation Medicine (ISPRM).

The search structure was prepared with the support of experienced librarians based at LSHTM and consisted of: (1) terms related to humanitarian crises/early recovery; and (2) terms related to public health interventions; and (3) terms related to low- and middle-income countries; and (4) terms related to injury and physical rehabilitation (see Electronic Supplemental Material). The reference lists for each of the selected articles were also reviewed in full to identify other relevant papers. Similarly, other reviewers participating in the R2HC-commissioned review were encouraged to recommend additional papers that were better suited to an alternative health topic (e.g. crush-related injuries captured during the non-communicable diseases search, which were more appropriately listed as injury and physical rehabilitation interventions).

### Paper selection and data extraction

**Fig. 1:**
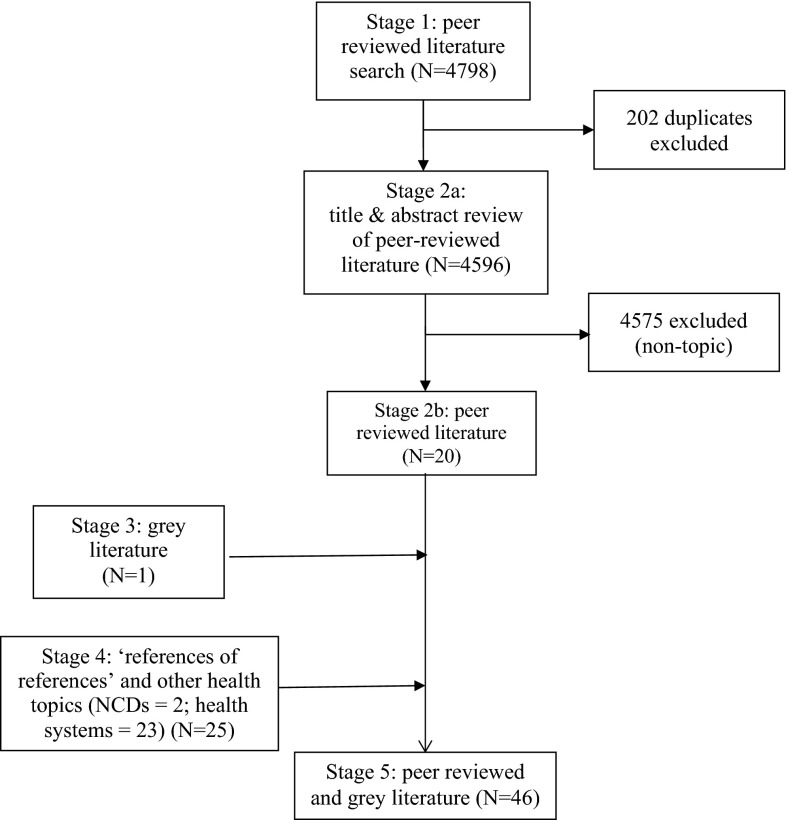
Screening process for the selection of papers

Papers were selected as part of a five-stage process, with one reviewer assessing the papers at each stage. The process was as follows: (1) the electronic database searches were performed and amalgamated; the results were imported into EndNote X6 reference software, and duplicate entries were removed; (2) papers were reviewed by title and abstract; (2a) manuscripts were reviewed in the event of ambiguity regarding the justification for inclusion or exclusion; (2b) studies were removed based on one or more of the exclusion criteria (see Table 1); (3) the grey literature sources were explored and again assessed against the inclusion and exclusion criteria; (4) the reference lists of selected papers were reviewed (‘references of references’) and additional papers captured during the concurrent health topic reviews were also assessed; (5) a final list of eligible papers was assembled, and data extraction and a quality assessment were performed in full (see Fig. 1). For quality assurance, a second reviewer corroborated study selection, data extraction, and study quality assessment.

Once selected, data from each of the papers were inputted into a Microsoft Excel database. The data captured included: study characteristics (i.e. author, year, study country, crisis setting); the study population (e.g. refugee, internally displaced, or general population); the nature of the humanitarian crisis (armed conflict or environmental disaster); the health outcome(s) assessed; the intervention evaluated; and the study methodology (e.g. study design).

### Study quality

Study quality was assessed using criteria distilled from an adapted version of the Strengthening the Reporting of Observational Studies in Epidemiology (STROBE) protocol (see Electronic Supplemental Material) (von Elm et al. 2007). The score range for the protocol was 0–8, with scores of 0–3 rated as low quality, 4–6 as moderate quality, and 7–8 as high quality. Studies were further categorised based on whether or not they reported a measure of statistical association. Those papers that quoted a statistical measure were graded A, while those papers that described the relationship between an intervention and a health outcome, but that did not quote a statistical measure were graded B.

## Results

### Search results

4798 studies were identified following a search of the peer-reviewed literature published between 1980 and 2013. A final total of 46 studies met the criteria for inclusion in this review (see Fig. 1). One paper was chosen from the grey literature search, while a further 24 papers were included following the non-communicable disease and health systems searches conducted during the commissioned, multi-topic evidence review.

### Crisis context

The majority of studies described programmes implemented during the acute phase of a humanitarian crisis. A total of eight studies assessed health outcomes during either the early recovery phase (Ebrahimzadeh and Rajabi 2007; Li et al. 2012; Motamedi et al. 1999; Roy et al. 2005), or the stabilisation phase (Tajsic and Husum 2008; Xiao et al. 2011; Zhang et al. 2012, 2013) (see Table 2). No papers were identified that examined the relationship between preparedness and health outcomes.

**Table 2:** Crisis context, population type, and study methodology

Study characteristics	%	*n*
Geographical region		
Asia	34.8	16
Eastern Europe	30.4	14
Middle East	26.1	12
Africa	4.3	2
Caribbean/Latin America	2.2	1
Multi-region	2.2	1
Crisis context		
Yugoslav wars (1991–1999)	30.4	14
Sichuan earthquake, China (2008)	21.7	10
Iran–Iraq war (1980–1988)	6.5	3
Iraq war (2003–2011)	6.5	3
Soviet war in Afghanistan (1979–1989)	6.5	3
Other	28.3	13
Crisis type		
Armed conflict	63.0	29
Environmental disaster	37.0	17
Population type		
General population	97.8	45
Refugee	2.2	1
Crisis location		
Urban	8.7	4
Rural	19.6	9
Mixed	71.7	33
Crisis phase		
Acute crisis	82.6	38
Early recovery	8.7	4
Stabilisation	8.7	4
Study type		
Cross-sectional	67.4	31
Longitudinal	21.7	10
Non-random trial	8.7	4
Economic	2.2	1

63 % of the studies documented health outcomes in situations of armed conflict, while the remaining 37 % assessed interventions implemented following an environmental disaster. The Yugoslav Wars of 1991–1999 were the most studied crisis setting, followed by the Sichuan Earthquake that devastated Wenchuan County, China in May 2008. A further three studies were published following each of the following crises: the Iran–Iraq war of 1980–1988 (Ebrahimzadeh and Rajabi 2007; Amirjamshidi et al. 2003; Gousheh 1995), the Soviet War in Afghanistan of 1979–1989 (Gosselin et al. 1993; Rautio and Paavolainen 1987; Strada et al. 1993), and the Iraq War of 2003–2011 (Fakri et al. 2012; Leininger et al. 2006; Zangana 2007).

Asia represents the most studied continent, of which the majority of studies originated from China. Eastern Europe and the Middle East were the source of 14 and 12 papers, respectively, while Africa and Latin America were particularly understudied geographical regions. A multi-country study in the Caribbean evaluated the cost-effectiveness of short, emergency orthopaedic programmes following the Haitian Earthquake in 2010, against a non-governmental organisation’s (NGO’s) established elective missions in neighbouring Dominican Republic (Gosselin et al. 2011). A single multi-region study examined the effect of traction versus external fixation for patients with high-velocity missile injuries treated at International Committee of the Red Cross hospitals in northern Kenya and Afghanistan (Rowley 1996).

### Study type

The majority of papers published adhered to a cross-sectional study design (*n* = 31), followed by a much smaller proportion of uncontrolled longitudinal studies (*n* = 10). Four studies utilised a non-randomised trial methodology: a joint Cambodian Ministry of Health and MSF study evaluated health outcomes following primary repair or colostomy for 102 war injured patients with penetrating intraperitoneal colon injuries (Moreels et al. 1994); one study by collaborating clinician-researchers at the Tehran University of Medical Sciences and Yale University School of Medicine examined the impact of a standardised rehydration protocol against existing hydration guidelines on the number of cases of acute renal failure, mortality, and the rate of fasciotomy among patients treated at three inner city hospitals following an earthquake in northwestern Iran in June 1990 (Nadjafi et al. 1997). A further two non-random trials, led by the same author, were published following the Sichuan Earthquake in 2008. The first study evaluated the functional health outcomes of 390 patients who had suffered fractures and subsequently received early, late, or no institutional rehabilitation (Zhang et al. 2012). The second study, published the following year, evaluated the physical functioning of patients who had received either early or late institutional and community rehabilitation against a control group that received neither institutional nor community rehabilitation (Zhang et al. 2013).

A single economic study compared the cost-effectiveness of the emergency relief operations of a small non-governmental organisation in Haiti and the Dominican Republic following the Haitian Earthquake of January 2010, against the organisation’s elective programmes in neighbouring Dominican Republic and Nicaragua in a similar time period (Gosselin et al. 2011).

### Trends in publication quantity and quality

**Fig. 2:**
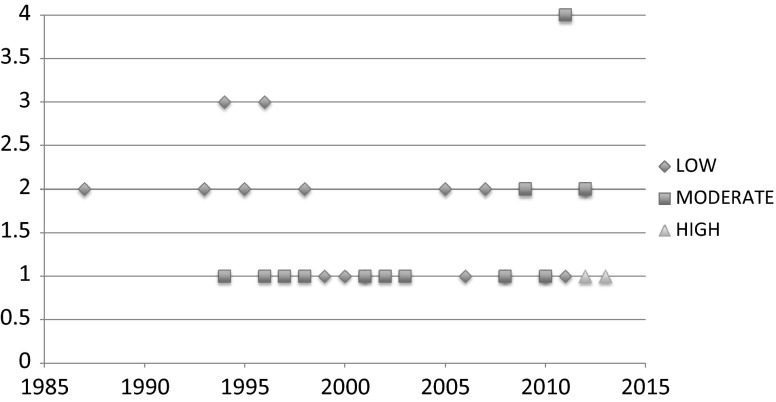
Number of studies published by year (1980–2013), disaggregated by quality

Both the quantity and the quality of papers have increased over the course of the last 33 years. Of the 46 studies, 58.7 % (*n* = 27) were published between the year 2000 and 2015. 79 % (*n* = 15) of the higher quality studies were published in the same time period (see Fig. 2).

Measured against the modified STROBE criteria, only 2 papers were considered of a high quality (Zhang et al. 2012, 2013). A further 17 papers were of a moderate quality, while the remaining 27 papers were deemed of a low quality. None of the papers met the full quality criteria, as sample size calculations were consistently absent.

Seventeen of the 46 papers evaluated health outcomes and quoted some form of significance test (category A). The remaining 29 articles described health outcomes following some form of surgical, medical, or rehabilitative intervention, but did not quote a statistical association (category B).

### Health outcomes and interventions

Orthopaedic injuries (*n* = 14), of which the repair of fractures featured prominently, were the most studied health outcome. Following orthopaedic outcomes, multiple or non-specific injuries featured frequently (*n* = 9), as did the medical and/or surgical response to crush injuries or renal failure (*n* = 7). Craniofacial injuries, and the repair of abdominal and thoracic injuries, were each the subject of five papers. A further three studies examined nerve or spinal cord injuries (Gousheh 1995; Li et al. 2012; Splavski et al. 1996), and three studies assessed revascularisation techniques or the repair of major blood vessels (Gosselin et al. 1993; Lovric et al. 1994; Roostar 1995).

Twenty-one studies described a range of non-specific surgical interventions. Seven papers looked at surgical external and internal fixation techniques in particular. This type of operation was the focal point of published research more frequently than any other complex surgical technique. Seven papers described different forms of renal therapy, and/or fasciotomy. A further four papers looked at health outcomes following limb amputation specifically (Ebrahimzadeh and Rajabi 2007; Fakri et al. 2012; Gosselin et al. 1993; Roostar 1995), while only four studies, all of which were carried out in China, evaluated different forms of rehabilitation (Li et al. 2012; Xiao et al. 2011; Zhang et al. 2012, 2013). Three papers examined pre-hospital care and triage (Bazardzanović et al. 1998; Jevtić et al. 1996; Roy et al. 2005).

## Discussion

This systematic review yielded 46 papers that assessed injury and physical rehabilitation interventions in humanitarian crises. Given that the review covered a 33-year catchment period, and in light of the heavy financial and human resource investment in emergency humanitarian operations during that period, these findings suggest that operational research remains the exception, rather than the norm.

From the available evidence, it is clear that the injury and physical rehabilitation sector is characterised by a strong focus on surgical and medical care; the rehabilitative needs of patients are markedly understudied. This is indicative of a preserved tendency toward short-term programming, with minimal or no follow-up in the post-acute phase of many humanitarian crises. These findings are not unique to injury and physical rehabilitation programmes; a lack of evidence for health interventions remains a cross-sectorial problem (Blanchet et al. 2013; Clarke et al. 2014).

The quality of studies remains highly variable. While there is arguably a trend towards an increased quantity and quality of research in recent years, many studies remain subject to methodological flaws; enrolment of a comparison group, adjustment for potential confounding factors, and justification of the study sample size were repeatedly absent from study methodologies. Such omissions are understandable given the rapidly developing nature of many humanitarian crises, and the reactive approach of many relief agencies. With this in mind, the available studies are representative of an opportunistic approach towards health research in humanitarian crises in recent decades.

Evidence gathering in the humanitarian sector remains a relatively new phenomenon for a number of reasons. Humanitarian programmes, particularly emergency surgical missions, during much of the twentieth century were short term and reactionary, with little or no prior planning or preparation beyond the need to provide immediate, lifesaving assistance. Insufficient population data in many crisis contexts also make it difficult for humanitarian agencies to identify target populations, and to situate research projects within a broader understanding of population health in any given context.

A promising drive towards population and donor accountability in humanitarian action, and the overarching moral obligation to provide the most effective and appropriate interventions in different crisis settings, has re-centred evidence-based care as an important programmatic objective. Recent studies suggest that decision-making in the humanitarian sector has been driven by organisational strategic priorities, established practice, and inter-agency relationships (ODI 2009; Darcy et al. 2013). The pursuit of evidence-based decision-making challenges this embedded behaviour, and encourages humanitarian agencies to reflect on the available evidence during the design and implementation of health programmes in crisis settings.

Following the launch of the R2HC programme in 2013, a number of studies have been funded in direct response to gaps identified by the health topic reviews (R2HC 2015). In light of the fact that research in crisis settings is often hindered by the unpredictable and rapidly changing nature of any given crisis context, recent calls for ‘off-the-shelf’ studies with full prior ethical approval may now help humanitarians to better integrate research alongside their existing programmes (Gerdin et al. 2014).

### Limitations

A number of limitations have affected this review. Foremost is the fact that the review looks specifically at research in humanitarian contexts. This is not to say that research conducted in stable settings does not carry value. While delivery mechanisms and variation in health needs are necessary considerations when comparing research generated in crisis and non-crisis settings, the well-studied benefit of certain interventions (e.g. fixation of fractured limbs) should not be overlooked.

Only English and French publications were selected for inclusion in this review. Given that a number of studies have emerged from China and the Middle East, it is possible that papers published in Mandarin and Arabic in national and regional journals have been overlooked. Similarly, as we did not capture Spanish or Portuguese publications, or search the LILACS database, our findings related to Latin America and the Caribbean should be viewed with caution.

### Conclusion

This review is the first of its kind to examine the quantity and quality of evidence for injury and physical rehabilitation interventions in humanitarian crises. While the evidence base has increased in recent years, inadequate attention has been paid to research in humanitarian settings as the number of humanitarian actors, and the budget allocated to humanitarian operations, continues to grow.

The trade-off between the need to act quickly and the need to act effectively presents a unique challenge for humanitarians. Humanitarian action can only benefit from the improved application of rigorously tested and context-appropriate research that identifies not only what works, but why. It is important not only to improve the quality of available evidence, but also to bridge the gap between the academic and operational communities. This will require a long-term vision, an iterative research process that is firmly embedded within new and existing systems for monitoring and evaluation, and a continuous dialogue between multiple stakeholders invested in the humanitarian endeavour.

## Electronic supplementary material

Supplementary material 1 (DOCX 97 kb)
