# Supplementary material for: A systematic literature review of the quality of evidence for injury and rehabilitation interventions in humanitarian crises
Source: Int J Public Health. 2015 Aug 23;60(7):865–72. doi: 10.1007/s00038-015-0723-6 (PMC4636531; doi:10.1007/s00038-015-0723-6)
Supplement: Supplementary file 1 — Supplementary material 1 (DOCX 97 kb) [file 38_2015_723_MOESM1_ESM.docx]

# Electronic SUPPLEMENTARY MATERIAL 1: Crisis, Context, and Study Search Terms

| exp Disasters |
| --- |
| exp Relief Work |
| Rescue Work |
| Emergencies |
| Emergency Medicine |
| Emergency Medical Services |
| Disaster Medicine |
| Mass Casualty Incidents |
| Emergency Responders |
| Medical Missions, Official |
| (humanitarian adj2 (crisis or crises or relief or response or agenc$)).tw. |
| humanitarian.tw. |
| (disaster adj3 (relief or plan$)).tw. |
| ((relief or aid) adj2 work$).tw. |
| Refugees |
| (refugee or evacuee or evacuated).tw. |
| (displace$ adj2 (force$ or population or human or internal$)).tw. |
| Altruism |
| exp War |
| war.tw. |
| ((armed or zone) adj2 conflict$).tw. |
| (conflict affected adj3 (population$ or person$ or communit$)).tw. |
| Avalanches |
| Earthquakes |
| Floods |
| Landslide |
| Tidal Waves |
| Tsunamis |
| Cyclonic Storms |
| (typhoon$ or hurricane$ or cyclone$).tw. |
| (avalanche$ or earthquake$ or flood or floods or flooding or flooded or landslide$ or tsunami$).tw. |
| (disaster adj2 (natural or victim)).tw. |
| Droughts |
| drought$.tw. |
| Starvation |
| (starvation or famine$).tw. |
| or/1-36 |
| randomized controlled trial |
| controlled clinical trial |
| cross-sectional studies |
| case-control studies |
| cohort studies |
| pilot studies |
| (random$ or controlled).tw. |
| (control adj3 (area or cohort? or compare? or condition or design or group? or intervention? or participant? or study)).ab. not (controlled clinical trial or randomized controlled trial).pt. |
| ((evaluat$ or prospective or retrospective) adj1 study).tw. |
| ("quasi-experiment$" or quasiexperiment$ or "quasi random$" or quasirandom$ or "quasi control$" or quasicontrol$ or ((quasi$ or experimental) adj3 (method$ or study or trial or design$))).tw. |
| ("time series" adj2 interrupt$).tw. |
| (intervention$ or impact or effectiveness or efficacy or service$ or outcome$ or output or treatment$ or management or program$ or project$).tw. |
| economics |
| cost-benefit analysis |
| cost control |
| Cost savings |
| cost of illness |
| cost $utility.tw. |
| (Cost$ adj2 effective$).tw. |
| cost-effective$.tw. |
| (cost adj3 utility).tw. |
| cost-utilit$.tw. |
| or/38-59 |
| developing countries |
| exp asia |
| exp africa |
| exp pacific islands |
| exp eastern europe |
| exp china |
| balkan peninsula/ or europe, eastern/ or transcaucasia |
| caribbean region/ or central america/ or "gulf of mexico"/ or latin america/ or south america |
| atlantic islands/ or indian ocean islands/ or macau/ or pacific islands/ or philippines/ or prince edward island/ or svalbard/ or west indies |
| or/61-69 |
| Japan |
| 70 not 71 |
| 37 and 60 and 72 |
| limit 73 to yr="1980 -2013" |

# Electronic SUPPLEMENTARY MATERIAL 2: Injury and Physical Rehabilitation Search Terms

Rehabilit* or physiotherapy* or prosthes* or orthes* or prosthetic* or orthotic* or crutch* or wheelchair* or orthopaedic* or disabled or physical* impair* or deficienc* or disabilit* or handicap* or cerebral pals* or spina bifida cystica or spina bifida occulta or muscular dystroph* or musculoskeletal abnormalit* or brain injur* or amputat* or clubfoot or poliomyelitis or paraplegia or hemiplegia or hearing loss or deaf* or blind* or vis* loss or intellectual disabilit* or learning disabilit* or developmental disabilit* or child development* disorder* or communication disorder*

# Electronic SUPPLEMENTARY MATERIAL 3: Modified STROBE and Protocol for Quality Assessment

| **Modified STROBE Criteria for Observational Studies** |
| --- |
| Intervention:  1. Is the intervention clearly described?  Selection of participants:  2. Is the target population defined?  3. Is there a comparison group (e.g. baseline, control)?  4. Are the inclusion and exclusion criteria defined?  Statistical methods:  5. Is the sample size / method justified with statistical basis?  6. Is there a statistical test (p-value or confidence interval)?  7. Is there adjustment for confounding?  Limitations:  8. Are study limitations explained (e.g. biases)? |

# Electronic SUPPLEMENTARY MATERIAL 4: Full EXTRACTION TABLE FOR SELECTED Papers

| **First author** | **Year** | **Study country** | **Humanitarian crisis type** | **Crisis stage** | **Health outcome(s)** | **Type(s) of health intervention** | **Study design** | **Modified-STROBE grade** | **Evidence category** |
| --- | --- | --- | --- | --- | --- | --- | --- | --- | --- |
| Amirjamshidi | 2003 | Iran | Armed Conflict | Acute crisis | Low velocity penetrating head injuries (disability, ability to work, mortality) | Minimal wound debridement (suture, referral) | Longitudinal | 4 | B |
| Bazardzanovic | 1998 | Bosnia and Herzegovina | Armed Conflict | Acute crisis | Craniocerebral injuries (outcomes post-surgery) | Application of first aid and medical care (e.g. exploration of wounds, treatment, antibiotics) | Cross-Sectional | 2 | B |
| Bumbasirevic | 2010 | Serbia | Armed Conflict | Acute crisis | Infected tibial nonunion after open fractures (refracture, infection, amputation) | Radical bony and soft tissue resection and bone transport - Iliza technique | Longitudinal | 4 | B |
| Chen | 2011 | China | Natural Disaster | Acute crisis | Gas gangrene infection (recovery, mortality) | Surgical treatment, quarantine antibiotics | Cross-Sectional | 2 | B |
| De Wind | 1987 | Uganda | Armed Conflict | Acute crisis | Missile injuries (healing, disability, union of fractures) | Surgical procedures in a resource limited setting | Longitudinal | 2 | B |
| Dediae | 1998 | Bosnia & Herzegovina | Armed Conflict | Acute crisis | Penetrating chest injuries (infection, mortality) | Thoracotomy, conservative treatment (thoracostomy and/or thoracocentesis) | Cross-Sectional | 4 | A |
| Dubravko | 1994 | Croatia | Armed Conflict | Acute crisis | Fracture of the extremities (complications, healing) | External fixation | Cross-Sectional | 2 | B |
| Ebrahimzadeh | 2007 | Iran | Armed Conflict | Stabilisation | Lower extremity injuries (pain, employment, psychological problems) | Foot / ankle amputation | Longitudinal | 3 | B |
| Fakri | 2012 | Jordan | Armed Conflict | Acute crisis | Infected and non-infected tibial non-union (recurrence of infection, readmission, non-union, mortality) | Amputation and / or reconstruction | Longitudinal | 4 | A |
| Gosselin | 2011 | Haiti & Dominican Republic | Natural Disaster | Acute crisis | Orthopaedic trauma (DALYs averted) | Orthopaedic surgery | Economic Study | 4 | B |
| Gosselin | 1993 | Pakistan | Armed Conflict | Acute crisis | Acute arterial injury – revasculisation (infection, pain, fistulae, contractures) | Amputation and / or revasculatisation procedures | Cross-Sectional | 3 | A |
| Gousheh | 1995 | Iran | Armed Conflict | Acute crisis | Brachial plexus injuries | Surgery (e.g. nerve grafts) | Longitudinal | 3 | B |
| Hammer | 1996 | Somalia | Armed Conflict | Acute crisis | Musculoskeletal injury of extremities (soft tissue recovery) | Unilateral external fixation device - hammer external fixation system (HEFS) | Cross-Sectional | 4 | B |
| Has | 2001 | Croatia | Armed Conflict | Acute crisis | Open fractures of the hands and feet (osteosynthesis, complications) | Minimal fixation method with Kirschner's wires | Cross-Sectional | 2 | B |
| Hudolin | 2005 | Bosnia & Herzegovina | Armed Conflict | Acute crisis | Penetrating colonic injury (complications, mortality) | Primary repair, colostomy | Cross-Sectional | 2 | B |
| Jevtic | 1996 | Bosnia & Herzegovina | Armed Conflict | Acute crisis | Range of physical injuries | First aid and evacuation | Cross-Sectional | 3 | B |
| Jiang | 2012 | China | Natural Disaster | Acute crisis | Range of physical injuries (discharge, mortality) | Medical, surgical and rehabilitation care (centralised treatment) | Cross-Sectional | 3 | B |
| Leininger | 2006 | Iraq | Armed Conflict | Acute crisis | Range of physical injuries (high energy) | Surgery, vacuum-assisted surgical dressings | Cross-Sectional | 3 | B |
| Li | 2012 | China | Natural Disaster | Early recovery | Spinal cord injuries (functionality, complications, independence in ADL) | Institutional rehabilitative programme | Longitudinal | 6 | A |
| Li | 2009 | China | Natural Disaster | Acute crisis | Crush injuries (improvement in bio-indicators, urinary output, mortality, amputation) | Intensive care unit (haemodialysis, prompt medical treatment) | Cross-Sectional | 5 | A |
| Li | 2011 | China | Natural Disaster | Acute crisis | Crush injuries (mortality) | Renal replacement therapy and blood transfusion | Cross-Sectional | 4 | A |
| Liu | 2010 | China | Natural Disaster | Acute crisis | Lower leg fracture (amputation, infection) | Orthopaedic surgery | Cross-Sectional | 2 | B |
| Liu | 2012 | China | Natural Disaster | Acute crisis | Tibial and fibular fractures (disability, complications) | External fixation and vacuum sealing | Longitudinal | 2 | B |
| Lovric | 1994 | Croatia | Armed Conflict | Acute crisis | Injury of major blood vessels of the extremities (mortality, reoperation, amputation, thrombosis) | Emergency surgical treatment (fasciotomy, antibiotics) | Cross-Sectional | 2 | B |
| Marcikic | 1998 | Croatia | Armed Conflict | Acute crisis | Penetrating craniocerebral injury | Surgery and management | Longitudinal | 2 | B |
| Moreels | 1994 | Cambodia | Armed Conflict | Acute crisis | Penetrating intraperitoneal colon injuries (complications, mortality) | Primary repair, colostomy | Non-Random Trial | 5 | A |
| Motamedi | 1999 | Iran | Armed Conflict | Early recovery | Maxillofacial injuries (complications, pain, functionality) | Branemark implant system | Longitudinal | 3 | B |
| Nadjafi | 1997 | Iran | Natural Disaster | Acute crisis | Acute renal failure (mortality) | Comprehensive diagnostic and treatment protocol | Non-Random Trial | 5 | A |
| Nikolic | 2000 | Serbia | Armed Conflict | Acute crisis | Missile injuries of the knee (complications, mortality) | Orthopaedic surgery, plaster cast, external fixation | Cross-Sectional | 3 | B |
| Ozturk | 2009 | Turkey | Natural Disaster | Acute crisis | Crush injuries – renal manifestations | Renal replacement therapy (continuous / intermittent haemodialysis) | Cross-sectional | 5 | A |
| Rautio | 1987 | Afghanistan | Armed Conflict | Acute crisis | Complicated fracture | External fixation, plaster cast, amputation | Cross-Sectional | 2 | B |
| Roostar | 1995 | Afghanistan | Armed Conflict | Acute crisis | Salvageable vascular injuries (amputation) | Amputation, reconstruction | Cross-Sectional | 2 | B |
| Rowley | 1996 | Afghanistan & Kenya | Armed Conflict | Acute crisis | Femoral, tibial, humoral fracture (shortening, hospital stay duration, complications) | Traction versus external fixation | Cross-sectional | 3 | B |
| Roy | 2005 | India | Natural Disaster | Stabilisation | Injury (infection rates, post-op outcomes, reoperations, psychological symptoms) | Field medical care and rehabilitation | Cross-sectional | 3 | B |
| Safari | 2011 | Iran | Natural Disaster | Acute crisis | Crush injuries (mortality, morbidity) | Fasciotomy | Cross-Sectional | 4 | A |
| Sagheb | 2008 | Iran | Natural Disaster | Acute crisis | Acute renal failure | Standard fluid therapy / variable volume treatment - dialysis | Cross-Sectional | 4 | A |
| Sever | 2002 | Turkey | Natural Disaster | Acute crisis | Acute renal problems | Renal replacement therapy | Cross-Sectional | 4 | A |
| Splavski | 1996 | Croatia | Armed Conflict | Acute crisis | Spinal cord injury (post-op complications) | Reconstructive surgery - laminectomy and dural repair (early surgery) | Cross-Sectional | 2 | B |
| Sprem | 2001 | Croatia | Armed Conflict | Acute crisis | Blast injury affecting the eardrum (successful healing) | Tympanoplasty (heterograft, temporal fascia, perichondrium) | Cross-Sectional | 6 | A |
| Stanec | 1994 | Croatia | Armed Conflict | Acute crisis | Physical injury (soft tissue lost, open fractures) (flap failure, reoperation) | Microvascular flap (local / free) reconstruction | Cross-Sectional | 3 | B |
| Strada | 1993 | Afghanistan | Armed Conflict | Acute crisis | Large bowel injury (wound infection, abscesses, mortality) | Surgical intervention with limited facilities (resection, anastomoses) | Cross-Sectional | 3 | A |
| Tajsic | 2008 | Balkan country (unspecified) | Armed Conflict | Early recovery | Landmine and blast injuries (complications, muscle functioning, healing) | Microsurgical post-injury reconstructive surgery (flap transfers) | Cross-Sectional | 2 | B |
| Xiao | 2011 | China | Natural Disaster | Stabilisation | Tibial shaft fractures | Rehabilitation intervention | Cohort | 6 | A |
| Zangana | 2007 | Iraq | Armed Conflict | Acute crisis | Penetrating liver injury | Emergency liver surgery | Cross-Sectional | 2 | B |
| Zhang | 2012 | China | Natural Disaster | Stabilisation | Bone fracture | Rehabilitation service programme (NGO-Health Sector-Volunteer) | Non-Random Trial | 7 | A |
| Zhang | 2013 | China | Natural Disaster | Early recovery | Physical disability (physical functioning) | Rehabilitation service programme (NGO-Health Sector-Volunteer) | Non-Random Trial | 7 | A |

# Electronic SUPPLEMENTARY MATERIAL 5: Full Reference List for Extracted Papers

1. Amirjamshidi A, et al. (2003), *Minimal debridement or simple wound closure as the only surgical treatment in war victims with low-velocity penetrating head injuries. Indications and management protocol based upon more than 8 years follow-up of 99 cases from Iran-Iraq conflict.* Surgical Neurology. Vol. 60(2): 105-10; discussion 110-1.
2. Bazardzanovic M, et al. (1998), *Craniocerebral injuries in combat soldiers treated at the Sapna war hospital, Bosnia and Herzegovina.* Croatian Medical Journal. Vol. 39(4): 446-9.
3. Bumbasirevic M, et al. (2010), *War-related infected tibial nonunion with bone and soft-tissue loss treated with bone transport using the Ilizarov method.* Archives of Orthopaedic & Trauma Surgery. Vol. 130(6): 739-49.
4. Chen E, et al. (2011), Management of gas gangrene in Wenchuan earthquake victims. Journal of Huazhong University of Science and Technology Medical Sciences. Vol. 31(1): 83-97.
5. de Wind CM (1987), *War injuries treated under primitive circumstances: experiences in an Ugandan mission hospital.* Annals of the Royal College of Surgeons of England. Vol. 69(5): 193-5.
6. Dedic SD, et al (1998), *Treatment of penetrating chest injuries during the 1992-1995 war in Bosnia and Herzegovina.* Croatian Medical Journal. Vol. 39(4): 442-445.
7. Dubravko H, et al. (1994), *External fixation in war trauma management of the extremities--experience from the war in Croatia.* Journal of Trauma-Injury Infection & Critical Care. Vol. 37(5): 831-4.
8. Ebrahimzadeh MH, Rajabi MT (2007), *Long-term outcomes of patients undergoing war-related amputations of the foot and ankle.* Journal of Foot & Ankle Surgery. Vol**. 46**(6): 429-33.
9. Fakri RM, et al. (2012), *Reconstruction of nonunion tibial fractures in war-wounded Iraqi civilians, 2006-2008: better late than never.* Journal of Orthopaedic Trauma. Vol. 26(7): e76-82.
10. Gosselin RA, et al. (2011), *Comparing the cost-effectiveness of short orthopedic missions in elective and relief situations in developing countries.* World Journal of Surgery, Vol. 35(5): 951-5.
11. Gosselin RA, et al. (1993), *Outcome of arterial repairs in 23 consecutive patients at the ICRC-Peshawar hospital for war wounded.* Journal of Trauma-Injury Infection & Critical Care. Vol. 34(3): 373-6.
12. Gousheh J. (1995), *The treatment of war injuries of the brachial plexus.* Journal of Hand Surgery - American Volume. Vol. **20**(3, pt. 2): s68-76.
13. Hammer RR, et al. (1996), *Simplified external fixation for primary management of severe musculoskeletal injuries under war and peace time conditions.* Journal of Orthopaedic Trauma. Vol. 10(8): 545-54.
14. Has B, et al. (2001), *Minimal fixation in the treatment of open hand and foot bone fractures caused by explosive devices: case series.* Croatian Medical Journal. Vol. 42(6): 630-3.
15. Hudolin T, Hudolin I (2005), *The role of primary repair for colonic injuries in wartime.* British Journal of Surgery. Vol. 92(5): 643-7.
16. Jevtic M, et al. (1996), *Treatment of wounded in the combat zone.* Journal of Trauma-Injury Infection & Critical Care. Vol**.** 40(3, supp.): s173-6.
17. Jiang J, et al. (2012), *Lessons learnt from the Wenchuan earthquake: Performance evaluation of treatment of critical injuries in hardest-hit areas.* Journal of Evidence-based Medicine. Vol. 5(3): 114-123.
18. Leininger BE, et al. (2006), *Experience with wound VAC and delayed primary closure of contaminated soft tissue injuries in Iraq.* Journal of Trauma-Injury Infection & Critical Care. Vol. 61(5): 1207-11.
19. Li CY, et al. (2011), *Continuous renal replacement therapy and blood transfusions in treating patients with crush syndrome: 8 Case studies from the Wenchuan earthquake.* Transfusion & Apheresis Science. Vol. 45(3): 257-60.
20. Li W, et al. (2009), *Management of severe crush injury in a front-line tent ICU after 2008 Wenchuan earthquake in China: an experience with 32 cases.* Critical Care. Vol. 13(6): r178.
21. Li Y, et al. (2012), *Evaluation of functional outcomes of physical rehabilitation and medical complications in spinal cord injury victims of the Sichuan earthquake.* Journal of Rehabilitation Medicine. Vol. 44: 534-540.
22. Liu L, et al. (2012), *The use of external fixation combined with vacuum sealing drainage to treat open comminuted fractures of tibia in the Wenchuan earthquake.* International Orthopaedics. Vol. 36(7): 1441-7.
23. Liu L, et al. (2010), *Treatment for 332 cases of lower leg fracture in "5.12" Wenchuan earthquake.* Chinese Journal of Traumatology. Vol. 13(1): 10-14.
24. Lovric Z, et al. (1994), *War injuries of major extremity vessels.* Journal of Trauma-Injury Infection & Critical Care. Vol. 36(2): 248-51.
25. Marcikic M, et al. (1998), *Management of war penetrating craniocerebral injuries during the war in Croatia.* Injury. Vol. 29(8): 613-8.
26. Moreels R, et al. (1994), *Wartime colon injuries: Primary repair or colostomy?* Journal of the Royal Society of Medicine. Vol. 87(5): 265-267.
27. Motamedi MH, et al. (1999), *Rehabilitation of war-injured patients with implants: analysis of 442 implants placed during a 6-year period.* Journal of Oral & Maxillofacial Surgery. Vol. 57(8): 907-13; discussion 914-5.
28. Nadjafi I, et al. (1997), *Suggested guidelines for the treatment of acute renal failure in earthquake victims.* Renal Failure. Vol. 19(5): 655-664.
29. Nikolic D, et al. (2001), *Missile injuries of the knee joint.* Injury. Vol. 31(5): 317-24.
30. Ozturk S, et al. (2009), The effect of the type of membrane on intradialytic complications and mortality in crush syndrome. Renal Failure. Vol. 31(8): 655-661.
31. Rautio J, Paavolainen P (1987), *Delayed treatment of complicated fractures in war wounded.* Injury. Vol. 18(4): 238-40.
32. Roostar, L. (1995), *Treatment plan used for vascular injuries in the Afghanistan war.* Cardiovascular Surgery. Vol. 3(1): 42-5.
33. Rowley D (1996), *The management of war wounds involving bone.* The Journal of Bone and Joint Surgery. Vol. 78 (5): 706-9.
34. Roy N, et al. (2005), *Surgical and psychosocial outcomes in the rural injured--a follow-up study of the 2001 earthquake victims.* Injury. Vol. 36(8): 927-34.
35. Safari S, et al. (2011), *Outcomes of fasciotomy in patients with crush-induced acute kidney injury after bam earthquake.* Iranian journal of Kidney Diseases. Vol. 5(1): 25-28.
36. Sagheb MM, et al. (2008), *Effect of fluid therapy on prevention of acute renal failure in Bam earthquake crush victims*. Renal Failure. Vol. 30(9): 831-835.
37. Sever MS, et al. (2002), *Treatment modalities and outcome of the renal victims of the Marmara earthquake.* Nephron. Vol. 92(1): 64-71.
38. Splavski B, et al. (1996), *Early management of war missile spine and spinal cord injuries: experience with 21 cases.* Injury. Vol. 27(10): 699-702.
39. Sprem N, et al. (2001), *Tympanoplasty after war blast lesions of the eardrum: retrospective study.* Croatian Medical Journal. Vol. 42(6): 642-5.
40. Stanec Z, et al. (1994), *The management of war wounds to the extremities.* Scandinavian Journal of Plastic & Reconstructive Surgery & Hand Surgery. Vol. 28(1): 39-44.
41. Strada G, et al. (1993), *Large bowel perforations in war surgery: one-stage treatment in a field hospital.* International Journal of Colorectal Disease. Vol. 8(4): 213-6.
42. Tajsic NB, Husum H. (2008), *Reconstructive surgery including free flap transfers can be performed in low-resource settings: experiences from a wartime scenario.* Journal of Trauma-Injury Infection & Critical Care. Vol. 65(6): 1463-7.
43. Xiao M, et al. (2011), *Factors affecting functional outcome of Sichuan-earthquake survivors with tibial shaft fractures: a follow-up study.* Journal of Rehabilitation Medicine. Vol. 43(6): 515-20.
44. Zangana AM. (2007), *Penetrating liver war injury: a report on 676 cases, after Baghdad invasion and Iraqi civilian war April 2003.* Advances in Medical and Dental Sciences. Vol. 1(1): 10-14.
45. Zhang X, et al. (2012), *Functional outcomes and health-related quality of life in fracture victims 27 months after the Sichuan earthquake.* Journal of Rehabilitation Medicine. Vol. 44(3): 206-9.
46. Zhang X, et al. (2013), *The NHV Rehabilitation Services Program Improves Long-Term Physical Functioning in Survivors of the 2008 Sichuan Earthquake: A Longitudinal Quasi Experiment.* PLoS ONE, 2013. Vol. 8(1).
